# Dissecting an alternative splicing analysis workflow for GeneChip^® ^Exon 1.0 ST Affymetrix arrays

**DOI:** 10.1186/1471-2164-9-571

**Published:** 2008-11-28

**Authors:** Cristina Della Beffa, Francesca Cordero, Raffaele A Calogero

**Affiliations:** 1 Department of Clinical and Biological Sciences, University of Torino, Regione Gonzole 10, Orbassano (TO), 10043, Italy; 2 Department of Information Technology, University of Torino, Corso Svizzera 185, Torino, 10149, Italy

## Abstract

**Background:**

A new microarray platform (GeneChip^® ^Exon 1.0 ST) has recently been developed by Affymetrix . This microarray platform changes the conventional view of transcript analysis since it allows the evaluation of the expression level of a transcript by querying each exon component. The Exon 1.0 ST platform does however raise some issues regarding the approaches to be used in identifying genome-wide alternative splicing events (ASEs). In this study an exon-level data analysis workflow is dissected in order to detect limit and strength of each step, thus modifying the overall workflow and thereby optimizing the detection of ASEs.

**Results:**

This study was carried out using a semi-synthetic exon-skipping benchmark experiment embedding a total of 268 exon skipping events. Our results point out that summarization methods (RMA, PLIER) do not affect the efficacy of statistical tools in detecting ASEs. However, data pre-filtering is mandatory if the detected number of false ASEs are to be reduced. MiDAS and Rank Product methods efficiently detect true ASEs but they suffer from the lack of multiple test error correction. The intersection of MiDAS and Rank Product results efficiently moderates the detection of false ASEs.

**Conclusion:**

To optimize the detection of ASEs we propose the following workflow: i) data pre-filtering, ii) statistical selection of ASEs using both MiDAS and Rank Product, iii) intersection of results derived from the two statistical analyses in order to moderate family-wise errors (FWER).

## Background

GeneChip^® ^Exon 1.0 ST is a new microarray platform developed and marketed by Affymetrix [1]. This microarray platform changes the conventional view of transcript analysis since it allows the evaluation of the expression level of a transcript by querying each exon component. This enables the study of specific alterations in splicing patterns such as those found in association with cancers [1].

The GeneChip^® ^Exon 1.0 ST microarray platform is based on methods quite different from the 3' IVT arrays expression detection. Whilst the conventional Affymetrix GeneChips feature a probe set consisting of 11–20 probes selected from the 3' end of the mRNA sequence, the new all-exon arrays have 4 probes selected from each putative exonic region. To generate the target, Exon 1.0 ST arrays use T7 linked random hexamers for cDNA synthesis, instead of those of all previous Affymetrix expression arrays, which employed an oligo-dT linked T7 and thus required an intact poly-A tail. Importantly, this new WT Sense Target Labeling Assay generates DNA targets and therefore results in DNA/DNA duplex formation during hybridization, as opposed to DNA/RNA hetero-duplexes in conventional arrays. It has been shown that there is close agreement between the conventional Affymetrix 3'IVT arrays and the new Exon 1.0 ST arrays [2]. Furthermore, Exon 1.0 ST sensitivity of gene expression detection was shown to be in the same range of 3'IVT arrays [3]. Though at gene-level 3'IVT and Exon 1.0 ST show similar behavior, Exon 1.0 ST technology raises some issues about the computational instruments to be used for the analysis of exon-level data. Affymetrix proposed an analysis workflow based on pre-filtering of the expression data (see Affymetrix technical documentation: id_altsplicingevents_technote.pdf), transformation of exon-level intensity data in gene-level normalized values called Splice Index (SI, see Affymetrix white paper: exon_alt_transcript_analysis_whitepaper.pdf) and statistical validation based on an ANOVA based method based on measuring differences between an exon-level signal and aggregated gene level signal called MiDAS (Microarray Detection of Alternative Splicing, see Affymetrix white paper exon_alt_transcript_analysis_whitepaper.pdf).

There has however been no way to date of defining the efficacy of this workflow or of different statistical methods in the detection of alternative splicing events. The ideal instruments to evaluate the effect of data pre-processing and the efficacy of different statistical methods on differential expressions are benchmark spike-in experiments [4], where a limited number of transcripts are spiked-in at various concentrations in a common mRNA background. In spike-in based experiments it is therefore possible to investigate differential expression sensitivity as a function of the false discovery rate (1-specificity). In this study a semi-synthetic exon-skipping experiment, encompassing 268 exon skipping events, was generated starting from the latin-square spike-in experiment of Abdueva [3]. The semi-synthetic exon-skipping data set was used to evaluate the effects of data pre-processing as well as the performance of two statistical methods, MiDAS and Rank Product (RP) [5], on ASEs detection.

## Results

### A benchmark experiment to validate ASEs detection methods

Exon skipping events were generated using the experimental data, kindly provided by Abdueva [3]. The Abdueva data set is a latin-square experiment encompassing 25 genes, selected as ideal spike-in genes due to their expression absence in HeLa cells, which represents the mRNA background of the experiment. The spike-in concentrations were 0, 2, 32, 128 and 512 pM and the 25 genes were grouped in 5 subsets. Each experimental point was technically replicated three times for a total of 15 arrays.

**Figure 1:**
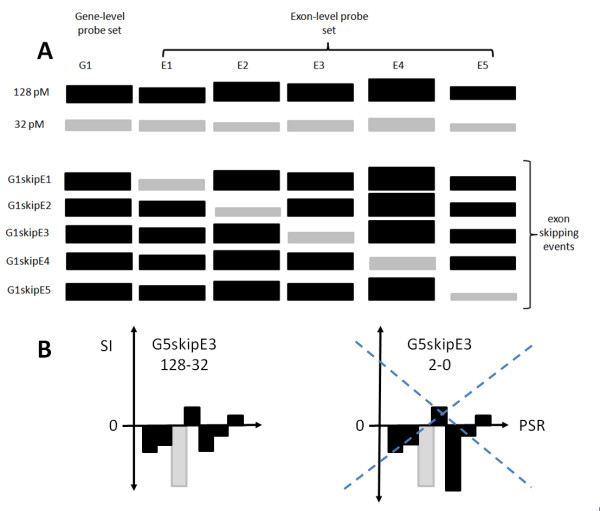
**Example of a set of exon skipping events and exon-skipping cleaning procedure**. A) Example of a set of exon skipping events. The gene-level probe set (gene) G1 is made of 5 exon-level probe sets (exons) E1, E2, E3, E4, E5. Exon-level probe set signals associated with 128 pM spike-in are black whereas signals associated with 32 pM spike-in are grey. New genes are created combining exon-level expressions derived from different spike-in concentrations. In this specific case, the combination of 128 and 32 pM spike-in signals for gene G1 are used for the generation of 5 new genes (G1skipE1, G1skipE2, etc) each one characterized by a skipping event, given by the spike-in at 32 pM, in one of the 5 exons of gene G1. The unspliced exons are instead given by the 128 pM spike-in. For the sake of simplicity only one out of the three technical replicates is shown. B) Exon-skipping cleaning procedure. The cleaning procedure, applied to all new genes characterized by a skipping event, retains only those where the synthetic skipping event represents the smallest intensity or SI value within the exons belonging to the gene. Here, it is shown the example of gene G5, which is made of 7 exons and therefore produces 7 new genes, G5skipE1, G5skipE2, etc. In G5skipE3 gene, exon E3 should be the only exon characterized by the smallest SI. G5skipE3 gene is retained in the set 128-32, since E3 (grey) is characterized by the smallest SI within all 7 exons (black). The gene is instead removed in the set 2-0 since exon E5 has a SI smaller than the one of exon E3.

To build the exon skipping benchmark experiment 4 out of the 5 groups of spike-in genes (20 out of 25 genes) were used. We focused on those because they were all part of the Exon 1.0 ST core annotation subset. The overall idea of the generation of synthetic exon skipping events is based on the availability of exon-level signals for spike-in genes. Therefore, it is possible to create new genes characterized by skipping events combining, for the same gene, exon-level expressions derived from different spike-in concentrations. An example is given in figure 1A, where the combination of 128 and 32 pM spike-in signals for gene G1 are used for the generation of 5 new genes each one characterized by a skipping event in one of the 5 exons of gene G1.

In our semi-synthetic data set the new genes, characterized by skipping events, are generated using different associations of spike-in concentrations to evaluate the effect of signal intensity in the detection of alternative splicing. For each exon of the 20 genes we produced three sets of synthetic exon skipping events: 128-32, 32-2, 2-0. Specifically in the exon skipping set called 128-32 any of the new genes has all exons signals given by the log_2_Intensity (log_2_I) measured upon a spike-in of 128 pM unless the exon skipped, which has the log_2_I measured upon a spike-in of 32 pM (fig. 1A, G1skipE1, G1skipE2, etc.). The gene-level log_2_I is instead the one measured for the 128 pM spike-in (fig. 1A). Same design applies to the other two sets of exon skipping events, 32-2 and 2-0.

This semi-synthetic benchmark experiment embeds a total of 268 exon skipping events. Furthermore, the skipping events were manually inspected, in each of the three exon-skipping sets (128-32, 32-2, 2-0), in order to retain only those genes where the skipping event represents the smallest intensity signal or Splice Index (SI is given by the log_2 _transformation of the ratio between each exon-level concentration and the corresponding gene-level concentration) within each synthetic gene (fig. 1B). This cleaning procedure yields:

• a total of 172 skipping events out of the original 268 for the 128-32 group, 195 for the 32-2 group and 179 for the group 2-0, if intensity data are used.

• a total of 174 skipping events out of the original 268 for the 128-32 group, 193 for the 32-2 group and 164 for the group 2-0, if SI data are used.

To identify exon-skipping events a comparison between two different conditions, i.e. unspliced versus spliced, is needed. Detection of exon-skipping events for the subset 128-32 was done comparing it to the unspliced set spiked-in at 512 pM (called 512), for the subset 32-2 comparing it to the unspliced set spiked-in at 128 pM (called 128) and for the subset 2-0 comparing it to the unspliced set spiked-in at 32 pM (called 32). These comparisons embed a certain level of differential expression at gene level. The expected gene-level differential expression is given by log_2_(128/512) = -2 for the comparison of the 512 versus the 128-32 subset and by log_2_(32/128) = -2 for the comparison 128 versus 32-2 subset. It is instead log_2_(2/32) = -4 for the comparison 32 versus 2-0 subset.

### RMA versus PLIER summarization

**Figure 2:**
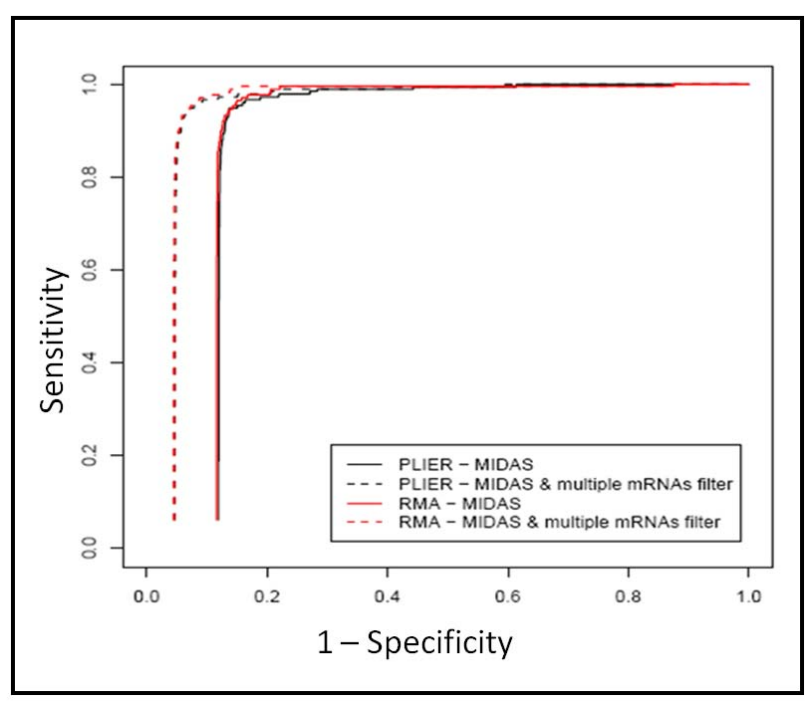
**MiDAS exon skipping detection using RMA or PLIER summarization**. ROC curves were used to identify the effect of data summarization on the detection of ASEs. ASEs were detected using MiDAS on the full core Exon 1.0 ST data set (continuous lines) using RMA (red line) or PLIER (black line). The same analysis was also applied to a subset of the core Exon 1.0 ST data set by encompassing only those gene/exon-level probe sets passing the multiple RNAs filter (dashed lines), i.e. those exons of genes associated to more than one mRNA isoform in ENSEMBL database.

RMA and PLIER algorithms were used to combine the intensities belonging to the probes of each probe set to form one expression measure for each gene/exon-level probe set (summarization). The effect of these summarization methods on detection of alternative splicing events was investigated using MiDAS. A Receiver Operating Characteristic (ROC) curve was used to evaluate the effect of intensity summaries on alternative splicing detection (Fig. 2, continue lines). Our data suggest that the efficacy of detecting exon skipping events is not affected by summarization methods. On the other hand the reduction of the complexity of the data set, e.g. selecting only those ENSEMBL [6] genes associated with more than one transcript isoform (multiple mRNAs filter), strongly increases the sensitivity of the test (Fig. 2 dashed lines).

### Filtering approaches to moderate multiple testing errors

A critical issue, highlighted in Fig. 2, is the important number of multiple testing errors that are accumulated if the full set of Exon 1.0 core data is used for the detection of ASEs. To moderate this critical issue, we decided to reduce the complexity of the data set filtering non-informative data (TN) before statistical analysis, using annotation and intensity based filters.

#### Cross hybridization filter

We investigated the effect of removing those probe sets characterized by a certain level of probe promiscuity among transcribed sequences (cross hybridization filter). Specifically, using the exon-level probe set annotation information provided by Affymetrix, we removed all probe sets where all the probes in the probe set perfectly match more than one sequence in the putatively transcribed array design content as well as those where the probes either perfectly match or partially match more than one sequence in the putatively transcribed array design content. This filter could have an important effect on the correct association of the gene expression signal. However, it affects a very limited number of exon-level probe sets and therefore it does not produce an important reduction of the size of non-informative data (Table 1). True Positives (TP), i.e. the semi-synthetic skipped genes previously described, are not affected by this filter since their exon-level probe sets are not annotated within the cross-hybridizing probe sets.

**Table 1:** Effect of annotation and intensity based filters on the selection of TP and reduction of unspliced exon set (TN).

	Splicing set		Splicing set		Splicing set	
	128.32 vs 512		32.2 vs 128		2.0 vs 32	
	TP (Sensitivity)	TN (Specificity)	TP (Sensitivity)	TN (Specificity)	TP (Sensitivity)	TN (Specificity)
Cross Hybridization filter	172 (1.00)	228264 (1.00)	195 (1.00)	228264 (1.00)	179 (1.00)	228264 (1.00)
Multiple mRNAs filter	172 (1.00)	71037 (0.31)	195 (1.00)	71037 (0.31)	179 (1.00)	71037 (0.31)
DABG filter (DABG p-value ≤ 0.05 in 90% arrays)	172 (1.00)	197951 (0.86)	185 (0.95)	197951 (0.86)	170 (0.95)	197951 (0.86)

The effects of filtering by means of annotation (Cross Hybridization/Multiple mRNAs filters) or intensity signal (DABG filter) are evaluated using exon-skipping events at various concentrations.

#### Multiple mRNAs filter

This filter uses the Affymetrix annotation that links each gene-level probe set to a specific GeneBank (GB) accession number (ACC), which represents the target sequence used to design the probes associated to a gene-level probe set. Then, Entrez Gene Ids (EGs) are retrieved querying with these ACCs a specific organism oriented Bioconductor  annotation package (org.Hs.eg.db, org.Mm.eg.db or org.Rn.eg.db). EGs are used to query ENSEMBL database and all ENSEMBL transcripts associated to any of them are retrieved. Subsequently, the filter procedure retains only those EGs associated to more than one ENSEMBL transcript. The EGs, retained by this filtering procedure, are mapped again to their gene-level probe sets.

Multiple mRNAs filter strongly reduces the number of core exons because it retains only exons of genes which are linked to multiple transcripts in the ENSEMBL database and for this reason it results to be more effective than the other filters as shown both in Table 1 and in Figure 2. The new genes, with skipping events, generated in our data set are not affected by this filter since they do not exist in nature.

#### DABG filter

In EXON 1.0 ST GeneChips, to determine if a given probe signal is detected above background (DABG), its intensity is compared to a distribution of background probes with the same G/C content. A p-value (DABG p-value) is computed representing the probability that the signal intensity is part of the null distribution. Specifically the DABG p-value filter, used in this work, is designed to retain only probe sets characterized by a DABG p-value ≤ 0.05 in all the arrays. Although this filter reduces the data set under analysis (Table 1), it is much less effective than multiple mRNAs filter (Table 1). Increasing the stringency of this filter affects the total number of non-informative data (TN), which is reduced, but also part of the TPs are lost. DABG p-values could be useful in the detection and removal of low intensity signals which could produce misleading results when alternative splicing events are evaluated using the Splice Index, where signal intensity component is lost to remove the bias due to the presence of gene-level differential expression. However, in our data set a filter based on this approach is much less effective than that based on multiple mRNAs filter (Table 1).

### Efficacy of MiDAS and Rank Product (RP) in the detection of alternative splicing events

We evaluated the efficacy of the detection of Alternative Splicing Events (ASEs), using a linear model based algorithm (MiDAS) and a permutation based algorithm (RP). MiDAS was applied on SI transformed data as RP was applied instead using SI, RP_SI_, or directly to exon intensity signals, RP_I_. RP was implemented both at gene-level and exon-level. Gene-level implementation of RP indicates that the analysis is performed gene by gene and the permutations are generated within the list of exons of the same gene. Exon-level implementation of RP considers instead exons as items of a unique list, independent from their association with a gene. The exon-level implementation of RP has better sensitivity than that of the gene-level version (data not shown) and is faster since permutations are calculated only once and not gene by gene.

**Figure 3:**
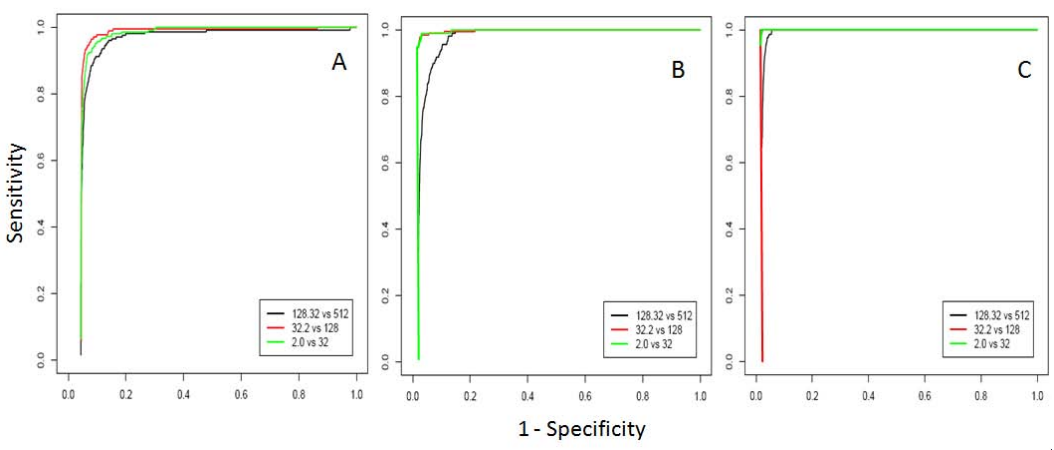
**Efficacy of MiDAS and RP in the detection of ASEs**. ROC curves were used to detect the efficacy of MiDAS and RP in the detection of ASEs. A) ROC curves for ASE detection using MiDAS. B) ROC curves for ASE detection using RP_SI_. RP was calculated using exon signal normalized with respect to gene signal, i.e. SI. C) ROC curves for ASE detection RP_I_. RP_I _was calculated using exon intensity signal without any further normalization.

Both MiDAS and RP seem to be effective in the detection of alternative splicing events independently from the presence of a certain level of gene differential expression and with limited dependency on gene-level intensity (Fig. 3). RP seems to perform slightly better than MiDAS. RP_I _(Fig. 3C) gives the most homogeneous results independently of the intensity signals associated with ASEs (Fig. 3B). Independently from the statistics in use, at p-value ≤ 0.05 (Table 2) the TPs detection is reasonably efficient for both methods, but is associated with a significant amount of False Positive values (FPs). We also evaluated, at the three intensity ranges under study, the number of TPs and FPs that can be detected intersecting all probe sets characterized by a p-value ≤ 0.05 both for MiDAS and RP (Table 2). The integration of the two statistical procedures improves the reduction of FPs without greatly affecting the sensitivity (Table 2).

**Table 2:** MiDAS and RP alternative splicing detection.

	Splicing set		Splicing set		Splicing set	
	128.32 vs 512		32.2 vs 128		2.0 vs 32	
	TP (Sensitivity)	FP (1-Specificity)	TP (Sensitivity)	FP (1-Specificity)	TP (Sensitivity)	FP (1-Specificity)
MiDAS	119 (0.68)	2416 (0.03)	176 (0.91)	2319 (0.03)	138 (0.84)	2338 (0.03)
RP_I_	174 (1.00)	12941 (0.18)	193 (1.00)	11883 (0.17)	164 (1.00)	9989 (0.14)
MiDAS & RP_I _intersection	119 (0.68)	436 (0.006)	176 (0.91)	424 (0.006)	138 (0.84)	375 (0.005)

RP_I _is the Rank Product calculated using the intensity signals without SI calculation. Statistical analyses done using MiDAS or RPI, calculated using intensity signals, at p-value ≤ 0.05 are contaminated by a significant number of FPs due to the multiple test problem. The intersection of the results using the two methods significantly reduces the number of FPs.

## Discussion

The availability of a new instrument to study the behaviour of transcription isoforms within a specific biological context, e.g. different cancer isolates, tissues, and differentiation/development stages, creates new opportunities for biologists. However, workflow for the detection of alternative splicing events using this new microarray technology has still to be investigated in order to define the importance of each analysis step and its strength and weakness. Our data point out that a major problem in ASEs detection is due to the multiple testing problem. In statistics, family-wise error rate (FWER) is the probability of making one or more false discoveries (FP) among all the hypotheses when performing multiple pairwise tests. Since FWER controlling procedures are often too conservative in high dimensional screening studies [7], they are rather weak if applied to exon-level analysis where the number of tests increases more than 10 times with respect to gene-level. For example, the human core data set is made of 22011 gene-level probe sets and 287329 exon-level probe sets. A better balance between the raw p-values and the stringent FWER-adjusted p-values may be provided by false discovery rate controlling and related procedures [7]. Benjamini and Hochberg [7] and Benjamini and Yekutieli [7] have developed efficient false discovery rate controlling procedures currently called BH and BY. However, such approaches cannot be used to moderate multiple testing problems in exon-level analysis since, generally, the raw p-value distribution obtained with MiDAS is not uniform in the non significant range. Furthermore, in the case of BH, the assumption that the tests are independent is not fulfilled since exons belonging to the same gene are clearly correlated. On the basis of the impracticality of applying conventional methods to moderate FWER, the reduction (filtering) of the data set size of previous statistical testing is, in our opinion, mandatory. Our data point out that a significant reduction of the data set size can be realized by considering only probe sets associated with at least two alternative spliced isoforms in the ENSEMBL database (multiple RNAs filter). However, this approach limits the strength of the analysis since it cannot be applied in the case of the identification of non-annotated isoforms. If a study focuses on the discovery of non-annotated isoforms, an intensity filter, e.g. DABG p-values filter, can be used although its effect is not as strong as that based on annotation (Table 1). In this case, it would be necessary to clean the results of the large amounts of false positives, validating data by using alternative technologies such as the high-throughput re-sequencing techniques, e.g. Solexa (Illumina, USA) or Solid (Applied Biosystems, USA). These would however increase the complexity of the analysis due to the high computational demands of these techniques. We also investigated the performance of two statistical methods, one based on linear model analysis (MiDAS), developed by Affymetrix for the detection of ASEs, and another non-parametric (RP). Both methods, applied at exon-level and thus not taking into consideration the association of an exon to a specific gene, perform quite well in the detection of the true exon skipping events embedded in our data set (Fig. 3). The amount of FPs associated to an arbitrary p-value threshold of 0.05 is in both cases very high (Table 2) and the application of a more stringent p-value threshold reduces the number of FPs but also impacts negatively on TP rate (data not shown). However, since the two statistics used for ASEs detection are based on completely different assumptions, it is feasible that random events (FPs) contaminating the TPs will not be the same. Therefore, the intersection of the results obtained by both statistics, given an arbitrary p-value threshold, effectively reduces FPs (Table 2). Since at the present time statistics specifically devoted to the detection of ASEs which also address the multiple test problem are not available, our approach represents an efficient temporary solution for moderating FWER.

## Conclusion

**Figure 4:**
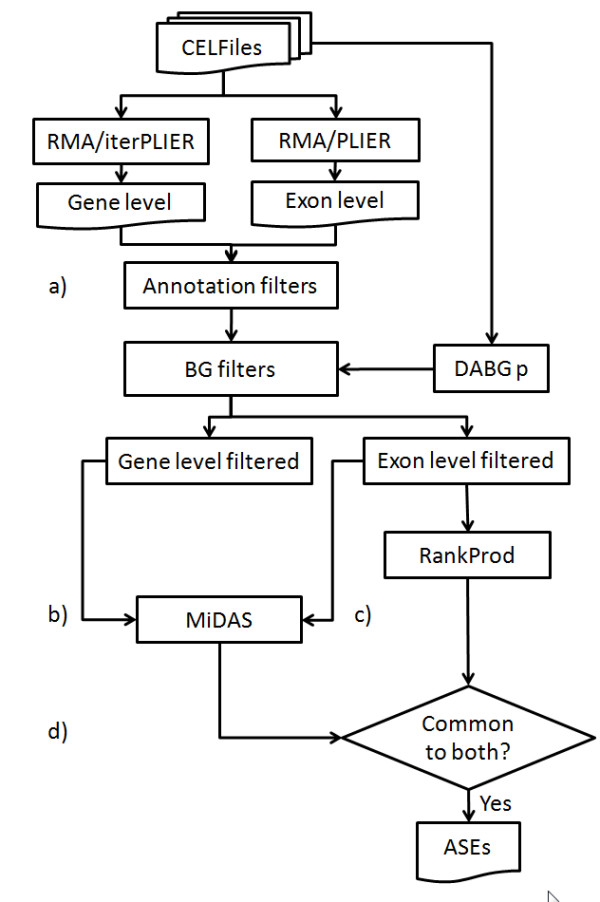
**Workflow for exon-level analysis**. Workflow proposed for the detection of ASEs. a) The number of probe sets to be considered for the analysis is reduced on the basis of ENSEMBL isoform knowledge (multiple RNAs filter). Eventually, a filter based on the quality of the intensity signal (DABG filter) might be considered as an additional filter. b-c) Statistical analysis is done using a model based algorithm (MiDAS) and a non-parametric algorithm (RP). d) Intersection of data derived by the two statistical analyses, using a common arbitrary p-value threshold (e.g. 0.05), is used to reduce the number of FPs.

The semi-synthetic data set presented here represents a suitable instrument for testing the efficacy of new statistics for exon-level analysis. Furthermore, it allowed us to test the efficacy of a basic workflow (Fig. 4) for ASEs using a GeneChip Exon 1.0 ST platform. However, our data highlights that more work is needed to design powerful instruments for ASE detection which must take into account the multiple testing problem.

## Methods

Exon-skipping events were generated using experimental data kindly provided by Abdueva [3]. MiDAS p-values were calculated using the software provided by Affymetrix in the APT tools . Rank Product (RP) is a non-parametric statistics that detects items that are consistently highly ranked in a number of lists and the significance of the detection is assessed by a non-parametric permutation test [5]. RP was coded in R , modifying the available implementation (Bioconductor [8] RankProd package), to be used for ASEs detection. Specifically, in ASEs detection RP is run on the lists made by SIs (RP_SI_) or intensities (RP_I_) for all exon data set without considering their association to a specific gene and the significance of the detection is assessed using 500 permutations of those lists. Gene-level implementation of RP, i.e. running RP only on the subset of exons belonging to a specific gene, is computationally demanding and it is characterized by a very poor sensitivity (data not shown).

The modified RP method as well as all the filtering procedures are embedded in Bioconductor oneChannelGUI [9] package. All scripts used for data generation and analysis are available upon request from the authors.

## Availability and requirements

Project name: Dissecting an alternative splicing analysis workflow;

Project home page: ;

Operating system(s): Platform independent, require at least 8 Gb RAM;

Programming language: R 2.7.0 , Bioconductor 2.2 .

Licence: The Artistic License, Version 2.0;

## Authors' contributions

CDB developed the benchmark data set and carried out the analyses described in this work. FC participated in the benchmark data set preparation. RAC supervised the study.
